# A Spoonful of Math Helps the Medicine Go Down: An Illustration of How Healthcare can Benefit from Mathematical Modeling and Analysis

**DOI:** 10.1186/1471-2288-10-60

**Published:** 2010-06-23

**Authors:** E Michael Foster, Michael R Hosking, Serhan Ziya

**Affiliations:** 1 Department of Maternal and Child Health, UNC Gillings School of Global Public Health, 407A Rosenau Hall, CB# 7445. Chapel Hill, NC 27599 USA; 2 Department of Statistics and Operations Research, UNC College of Arts & Sciences, 319 Hanes Hall, CB# 3260. Chapel Hill, NC 27599 USA

## Abstract

**Objectives:**

A recent joint report from the Institute of Medicine and the National Academy of Engineering, highlights the benefits of--indeed, the need for--mathematical analysis of healthcare delivery. Tools for such analysis have been developed over decades by researchers in Operations Research (OR). An OR perspective typically frames a complex problem in terms of its essential mathematical structure. This article illustrates the use and value of the tools of operations research in healthcare. It reviews one OR tool, queueing theory, and provides an illustration involving a hypothetical drug treatment facility.

**Method:**

Queueing Theory (QT) is the study of waiting lines. The theory is useful in that it provides solutions to problems of waiting and its relationship to key characteristics of healthcare systems. More generally, it illustrates the strengths of modeling in healthcare and service delivery.

Queueing theory offers insights that initially may be hidden. For example, a queueing model allows one to incorporate randomness, which is inherent in the actual system, into the mathematical analysis. As a result of this randomness, these systems often perform much worse than one might have guessed based on deterministic conditions. Poor performance is reflected in longer lines, longer waits, and lower levels of server utilization.

As an illustration, we specify a queueing model of a representative drug treatment facility. The analysis of this model provides mathematical expressions for some of the key performance measures, such as average waiting time for admission.

**Results:**

We calculate average occupancy in the facility and its relationship to system characteristics. For example, when the facility has 28 beds, the average wait for admission is 4 days. We also explore the relationship between arrival rate at the facility, the capacity of the facility, and waiting times.

**Conclusions:**

One key aspect of the healthcare system is its complexity, and policy makers want to design and reform the system in a way that affects competing goals. OR methodologies, particularly queueing theory, can be very useful in gaining deeper understanding of this complexity and exploring the potential effects of proposed changes on the system without making any actual changes.

## Introduction

Over the past two decades, operations researchers increasingly have examined health care systems. One of the leading journals in the field, *Operations Research*, devoted an entire issue to health care research in November, 2008 [1]. This research employs the latest in operations research methodology (e.g., Ross and Jayaraman [2]). Articles published in the operations research (OR) literature examines a broad range of issues, including (but not limited to) capacity planning and management in hospitals [3,4] and multisite service systems [5]; organ donation and allocation [6,7] and dialysis [8]; workforce scheduling [9]; the occurrence of disease, including mental disorder [10]; the effect of promotional tools [11]; patient queues and delays [12,13]; the prediction of health care costs [14]; drug treatment [15]; the effects of reimbursement policy [16,17]; and breast cancer diagnosis and treatment[18].

In contrast, very little of this research appears in the standard journals in health policy and health services research (for exceptions, see [19-21]). The disjuncture, therefore, lies between the development of these tools and their application to real-world problems. This need is reflected in a recent joint report from the Institute of Medicine and the National Academy of Engineering. This landmark report identifies many potential benefits of OR in healthcare and recommends several measures to strengthen the link between the two. For example, the report recommends that health care become one of the standard applications taught to engineering students. Conversely, the report advocates that providers integrate system tools in the actual delivery of care. Such tools might include system-wide data standards and hand-held digital recall devices for doctors and nurses.

An OR perspective typically frames a complex problem in terms of its essential mathematical structure. Such a model has three main components: an objective function, decision variables, and constraints. The purpose of the model is to identify the relationships between alternative choices and key outcomes. For example, a common application of OR tools involves queues for services. In a typical queueing application, the objective could be to minimize staffing costs, a constraint could be that average waiting time remains below some level, and the decision variable could be the number of servers to be employed. Once the model is specified, OR offers a variety of tools for understanding the implications of alternative choices. For example, a mathematical solution may identify the optimal decision and allow one to estimate the impact of sub-optimal choices. In many instances, standard mathematical solutions for common problems exist; many rather different applications (e.g., the line at a bank teller or the waiting room at a health clinic) have a similar mathematical structure.

Like any modeling, OR simplifies the actual phenomena. A model generally cannot completely represent every detail of a complex system. A model captures the essence of a system; as a result, some details of that system are ignored. One needs to balance the level of detail with the analytic tractability of the model. As model complexity grows, the model becomes more realistic yet more difficult to solve. Standard solutions may no longer exist, requiring the analyst to develop complex simulations or new mathematical techniques to solve the problem. One can balance these concerns by calibrating the model--by assessing its ability to reproduce key features of the system(s) being modeled.

Modeling has several benefits. The model may identify unanticipated, system-wide consequences of a decision. For example, adding more lines at a fast food restaurant during lunch time may generate increasingly small reductions in waiting time by customers; or laying off one of our four cashiers may increase waiting times only by 10%. Particularly valuable is the fact that the model may reveal these consequences before the decision is actually implemented.

This article illustrates the use and value of operations research tools in health care. We employ queueing models as an illustration. Queueing models are useful in that they provide solutions to problems of waiting that are particularly relevant in health care. More generally, they illustrate the strengths of modeling in health care research and service delivery. Section 1 provides background on queueing theory. Section 2 provides some examples of how queueing theory has been used in healthcare. Section 3 develops a modeling approach for an illustrative example, drug treatment. This section also makes a broader point involving the benefits of modeling more generally.

## Methods: A Brief Introduction to Queueing Theory

Queueing Theory (QT) is the study of waiting lines and is one of the oldest areas of OR. QT grew out of an article by Erlang [22], which provided some of the most widely used tools in mathematical modeling. QT focuses on systems in which "customers" arrive, wait for their turn for service, are served, and then leave. Telecommunications, inventory management, and healthcare all represent areas of application to which these tools have been applied.

Queues develop because of the random manner in which customers arrive and the times it take to serve them. In many systems, administrators need an understanding of the relationships between key performance measures and controllable system parameters. QT defines these relationships mathematically under certain conditions, which reveals the potential effects of decisions on performance measures. For example, the theory allows one to determine a mathematical expression that relates average customer waiting time to the number of servers. The decision-maker then can examine how the average waiting times change with the number of servers and determine how many servers to employ accordingly.

Queueing theory offers key insights that initially may be hidden. For example, a formal model allows one to incorporate randomness in key parameters, such as arrivals of phone calls at a system. As a result of this randomness, these systems often perform much worse than one might have guessed based on deterministic conditions (no randomness). Poor performance is reflected in longer lines, longer waits, and conversely lower levels of server utilization overall. For example, an emergency room may have sufficient staffing for the average load on a night, but the staffing level will not be sufficient on all nights because of the randomness in patient arrivals. As a result some of the patients will end up waiting for a very long time. QT can help in determining staffing levels that will help ensure that the percentage of long waits remain below a certain level.

Most queues we observe in our daily lives may seem simple from the outside, but they are in fact so complex that they cannot be characterized mathematically. For example, we can easily determine the arrival rate of cars to a drive-thru fast-food restaurant, but a complete mathematical description of the exact times at which cars will arrive is mathematically difficult. Even in cases where such characterization is possible, the description is typically very complex and therefore does not permit mathematical analysis. Any QT work assumes the existence of certain conditions on the system analyzed, which conveniently allow the mathematical analysis to proceed. Thus, QT analyzes "idealized" models, which typically do not exist in practice but can serve as approximations ranging from reasonable to excellent. Often, the same idealized model can be used to represent a variety of queueing systems, e.g., one model may approximate the ticket line at a movie theater, the cars lined up at the drive-thru, and the patients waiting in the emergency room.

Queueing theorists typically use Kendall's notation as short-cut notation for complete descriptions of queueing models. (See Table 1.) That notation comprises five essential characteristics. These are the (1) Arrival Process (A), (2) Service Time Distribution (B), (3) Number of Servers (C), (4) System Capacity (K), and (5) Service Discipline (D). The first characteristic, the arrival process, generally is specified as deterministic or stochastic. Deterministic processes involve constant times between events (such as customer arrivals); stochastic processes involve random variation in these times. The last characteristic refers to the process that determines the order in which waiting customers are served. The queue is then simply described (By Kendall's notation) as the "A/B/C/K/D queue." If the system capacity K is not given, it is assumed to be infinite. If the service discipline is not given, it is assumed to be First-Come-First-Served (FCFS).

**Table 1:** Kendall's notation for Queueing Models

Position	Meaning	Description
1^st ^(A)	Arrival Process	This parameter describes how customers arrive at the system. In particular, whether they arrive in groups or as individuals and the distribution of inter-arrival times.

2^nd ^(B)	Service Time Distribution	This parameter describes the distribution of service times.

3^rd ^(C)	Number of Servers	Often this parameter is 1, meaning that there is only one server, but multi-server systems are common, and so most results are generalized to an arbitrary number of servers, c. Some queues can also have infinite servers.

4^th ^(K)	System Capacity	This parameter indicates how many custometers can be served at one time, including those in service. It is often assumed to be sufficiently large as to never be an issue.

5^th ^(D)	Service Discipline	This parameter refers to the order (or discipline) that arriving customers are served. For most examples the discipline is First Come First Served (FCFS). But other options exist such as Last Come First Served (LCFS) and Service In Random Order (SIRO)

For example, the notation M/D/3/20 indicates a queueing system in which arrival process is Markovian (inter-arrival times are exponentially distributed); service times are deterministic; there are three servers; system capacity is 20; and service discipline is FCFS. On the other hand, D/G/1 queue has deterministic arrivals, general service times (i.e., service time distribution is irrelevant), single server, infinite system capacity, and FCFS as the service discipline.

The QT literature offers many standard queueing models. The solutions to those models reveal its key features, such as mathematical expressions for the average waiting time. The results of these analyses can be readily used in different application areas where these queueing models are good fits. For example, one of the simplest queueing models is the M/M/1 queue. For this queue, if the arrival rate is λ, and the expected service time is 1/μ, the average number of customers waiting in the system (including those receiving service) is (λ/μ)/(1 - λ/μ). Then, if one is interested in determining the average number of customers waiting in a queueing system for which the M/M/1 queue would be a good fit, all s/he needs to do is plug in the actual values for the arrival rate λ, and the service rate μ. QT provides similar ready-to-use solutions to a number of queueing systems although the mathematical expressions can be significantly more complex.

QT literature is not limited to models that can be described by Kendall's notation. There are many realistic features that one can add to such models although the analysis typically gets more complex with each addition. For example, one feature relevant to healthcare is *reneging customers *. These customers choose to leave the system before being served. "Leaving" can describe a wide range of phenomena within the healthcare setting. In an emergency room leaving can be those who get tired of waiting. On the other hand, in a mass casualty event, leaving could refer to those patients who die before being seen. Another set of models that are relevant within healthcare setting are queueing networks. In these models customers move between queues and possibly leave the system at some point. Such models can describe the movements of the patients, for example, between surgery, the recovery room and regular inpatient care [23].

While vast, the QT literature does not provide answers for every possible queueing model. In general, as one changes the Markovian assumptions for the arrival and service processes or adds some of the complications discussed above, the analysis becomes more complex. For such systems, simulation may be better suited. In a simulation study, researchers first build a model of the actual system using simulation software. When simulating, the computer generates vast numbers of customers (or other entities) who travel through the model, which could consist of a single queue or a network of queues. As these entities travel through the model, the computer records the desired data, which are then used to describe system performance, such as the average waiting time per customer.

Simulation is one tool for bypassing the difficult analytical problems resulting from complex queueing situations. Another involves mathematical approximations to simplify features of the model (such as the objective function). In most interesting problems, difficult equations arise which are impossible to solve generally. However, various approximations can make the problem tractable (e.g., a Taylor Series approximation to a difficult-to-evaluate function).

### Applications of Queueing Theory in Health Care

Queueing models have been used to answer a variety of questions in health care. These applications involve a range of problems that vary greatly in scale. Some examples are how to allocate hospital beds [24], schedule surgeries [25], and triage patients [26], but researchers typically have focused on waiting times and utilization. These papers have explored the trade-offs between these two goals, minimizing patients waits and maximizing resource (staff, equipment, beds, etc.) utilization. Green [27] provides a good overview of this literature. Healthcare applications tend to be very complex, and so there have been a number of extensions to the classic models to bridge the gap between the models and reality. Some extensions include reneging (i.e., leaving the queue before being served) [28], variable arrival rates [29], and blocking (i.e., customers done with one phase of service, not advancing through the system due to others being served) [30].

Some QT research has examined patient flows and considered how redistribution of resources or redesign of systems could improve flow. Often commercial software, such as QNA, is very helpful to real-world providers. Such applications may reduce or eliminate bottlenecks that reduce quality of care [31].

Queueing has also been used in the design of whole systems in healthcare. Most of this work focuses on finding proper capacity, such as the marginal cost of additional beds [32]. Some studies examine multiple elements working together such as facilities working in the same region [30,33].

The next section illustrates queueing theory and the tools of OR more generally. This section will look at how a simple model in QT can answer difficult questions in healthcare management.

## An Illustration: The Management of Drug Treatment Facilities

The history of treatment for substance abuse is long, and effective drug treatment has proven elusive. Some programs have demonstrated success. Brief intervention and social skills training have both shown significant efficacy (in particular over traditional 12-step programs, [34]). *Simply finding an effective treatment, however, is only the beginning of service delivery *. A range of problems separate potential patients from actually receiving appropriate care. These issues include funding as well as training providers to actually deliver the service. Key issues of capacity planning also are involved: a provider offering the effective treatment needs to deliver it in sufficient quantity to those who would benefit.

For these types of problems, OR in general and queueing models in particular have much to offer. Consider a residential drug treatment facility. This hypothetical facility can be modeled using an M/D/c queue. This model was chosen because it best approximates many key features of this application. First, potential customers (individuals requiring treatment) arrive, at random, according to a Poisson process with some constant rate (M for arrivals). If space is available for them to enter treatment they do; otherwise, they must wait. Second, services take a deterministic amount of time: each patient spends k weeks in treatment and then is discharged (D for service). Facility capacity is determined by the number of patients they can house at one time, here referred to as the number of beds (c servers). We also assume that the waiting list has no maximum and that the arrival rate does not depend on the number of clients being treated at a point in time. Finally, we assume decisions about whom to treat is "First Come First Served".

Tijms [35] has examined this specific queue. The solution involves solving an infinite set of linear equations. The author does so by using a common approximation in OR: he reduces the number of equations to a finite number using the geometric tail approximation. This approximation assumes that the probability of having successively greater numbers of people in a queue decays exponentially as the length of the queue increases. Using this simplified system, one can solve directly for the long-run average number of people in the queue. From that solution, one can calculate other key outcome measures, such as the distribution of waiting times, the proportion of arrivals that need to wait and measures of system utilization. (See mathematical appendix 1 for more details. The appendix also provides the Matlab code needed to produce the solutions reported below.)

One can take Tijms' results and examine questions about performance or questions policy maker might have regarding the facility. For example,

• What will average occupancy be? How long can patients expect to wait for a bed?

• Can the facility accommodate referrals from a local hospital? If so, how many extra beds will be required?

• If this facility is consolidated with two others (leaving overall capacity unchanged), who will benefit? Customers, the facilities, both or neither?

To answer these questions, one needs the 3 key parameters. Any facility can provide these figures; we selected hypothetical values for arrival rate (λ = 1 per day); for length of treatment (28 days; μ = 1/28); and number of beds (C = 32). We illustrate the model's solution for these hypothetical parameters, but of course, the reader with access to Matlab could calculate a solution for a different facility, perhaps better reflecting the circumstances in his or her community.

**Question 1**: What will average occupancy be? How long can patients expect to wait for a bed? 

The long-run expected occupancy at a point in time is 28 beds occupied. On 33.6% of all days, the facility is full; 66.4% of patients experience no wait for services. Only on 22.2% of days are fewer than 25 beds occupied. Of those who do wait, the expected wait for a bed is 4.11 days. Only 5.8% of patients have to wait more than one week. So, based on these benchmarks our hypothetical clinic is meeting realistic operating goals.

**Question 2: **Can the facility accommodate referrals from a local hospital? If so, how many extra beds will be required? 

Suppose a local hospital is considering closing its treatment unit. If it does, it will refer an average of 2 patients per week. Based on the hospital's estimate, the arrival rate will now be 9/7 (1.2857) patients per day. We know that not all beds are in continual use now, since the occupancy should be 28 patients, so we might try adding no beds. However, from an easy check for stability we see that will not work. From introductory QT, it is well known that if there is no limit to the number waiting and λ ≥ μ * *C*, the system is unstable (the length of the line will grow without bound). Put into words, if the arrival rate is greater than the total service rate when all servers are working, the facility will never (in the long run) be able to keep up. We can clearly see from the stability condition we need at least 36 beds (additional 4 beds)

**Figure 1:**
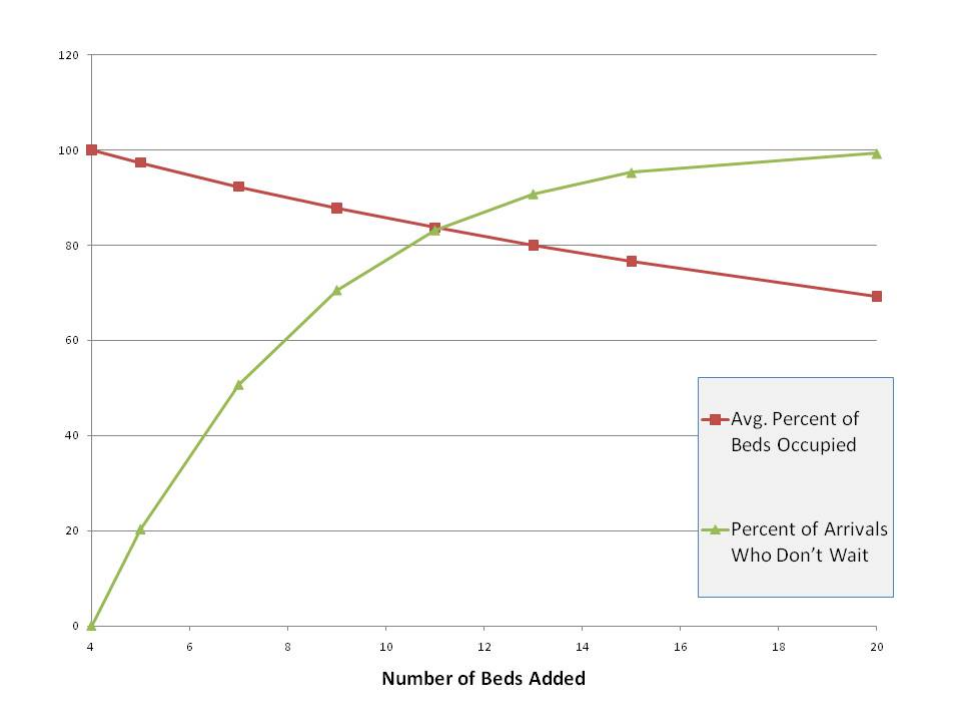
**Number of beds added and key performance measures**.

**Figure 2:**
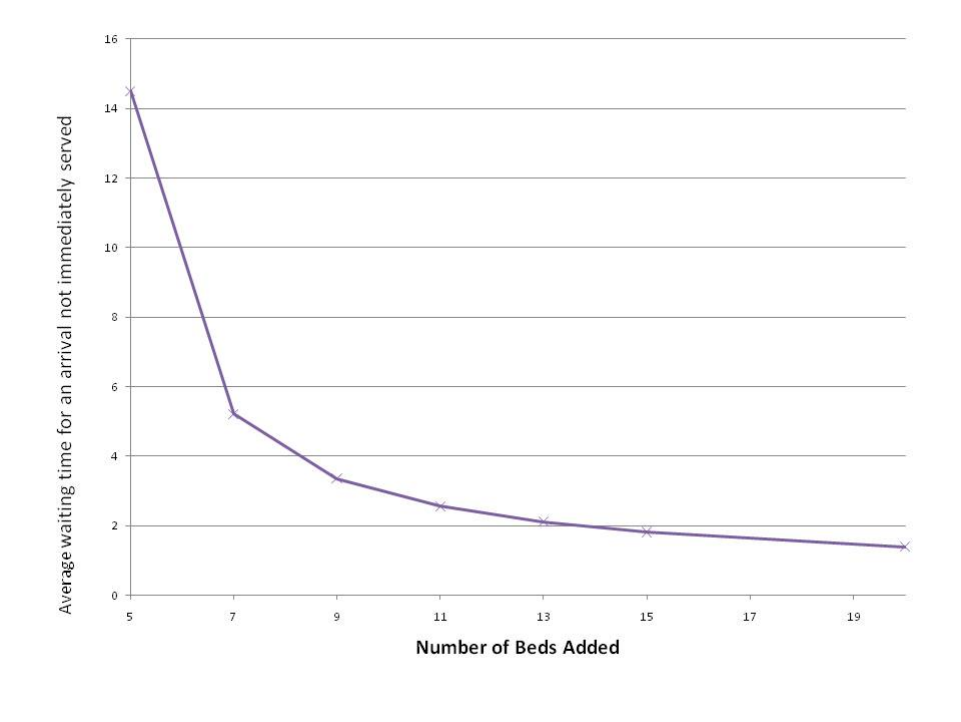
**Average waiting time for an arrival not immediately served**.

How many beds to add beyond 4 is difficult to calculate because one must decide how much of an increase in waiting is tolerable. However, by utilizing QT one can look at the various possibilities instantly; one can determine the various outcomes without having to implement them and then observe the consequences. Figures 1 and 2 report the relationship between added beds and waiting times.

Figure 1 shows the relationship between the number of beds added and two key performance measures. At 7 beds, the average percent of beds occupied is 92.3%, a slight increase over the original rate (87.5%). 9 additional beds restore the system to this original rate. At 7 beds, roughly half of arrivals do not wait (50.7%)--a rate substantially worse than observed originally (66.4%).

Figure 2 reports the average waiting time for an arrival that is not immediately served. For 7 beds, the wait is marginally longer than our original model (5.21 days). At 9 beds, the impact of the additional customers is eliminated.

From the figures it is clear that we should add more than 7 beds, otherwise we expect performance metrics to deteriorate, at least from the client's perspective. From the facility perspective, some benefits accrue: the percent of beds occupied increases. Adding 9 beds keeps the performance metrics at similar (or slightly improved values) at a minimum number of additional beds.

**Question 3: **Suppose this facility is one of three in the area and that the owners of the facilities are considering a merger under which patients would be shared across facilities. Who will benefit? Patients, the firms, both, or neither?

Assuming that the two other facilities are identical to our initial facility (1 arrival per day, 28 day treatment, and 32 beds) and close enough such that patients could be moved from one facility to the other with negligible cost, the three merged facilities would have an overall capacity of 3 arrivals per day, offers 28 days of treatment, and administers 96 beds. Does tripling both the number of beds and customers produce any real changes in the functioning of the facility? While not apparent, this merger (or pooling) does fundamentally change the nature of the system. Now excess capacity in what used to be a separate facility can be shared with the other facilities. What does this change mean for key stakeholders?

After the merger the average occupancy is unchanged (87.5%). However, the patients' experiences are dramatically improved. The percent of arrivals who experience no wait rises to 87.7% (from 66.4%) and the expected wait drops to 1.55 days (from 4.11). clearly this shows that the merger should improve the experiences of patients.

The owners of the firm will be interested in how they would benefit from the merger. In the post-merger system, while the average occupancy has stayed the same, the number of idle beds has increased. To raise revenue, the facility may take more transfers from other facilities or otherwise increase the number of patients without damaging the patients' experience.

Table 2 reveals the effect of the additional arrivals. The first line shows the key system characteristics at the original arrival rate. We see that small increases in the arrival rate cause the occupancy to increase substantially. Increasing arrivals to 3.3 per day (a 10% increase) raises occupancy to 96.3%. Concomitantly, the patients' experience deteriorates--now only 41.4% are admitted immediately, and the waiting times (for those who do wait) increases to 4.24 days. Further increases are especially dramatic. At 3.5 arrivals, all of the beds are occupied all of the time, and all patients wait. And the system is now unstable as waiting times explode without bound.

**Table 2:** Effect of the additional arrivals on Key Outcomes

Arrivals rate (per day)	Avg. Percent of Beds Occupied	Percent of Arrivals Who Do not Wait	Avg. Wait Given That the Arrival Must Wait (in days)
3, (After Merger, no action)	87.5	87.7	1.55

3.1	90.4	78.0	1.90

3.2	93.3	63.2	2.55

3.3	96.3	41.4	4.24

95/28 (3.39)	99.0	13.2	14.3

3.5 and above	100	0	Infinite

Original situation (pre merger)	87.5	66.4	4.11

An alternative option would be to cut the number of beds and so reduce overhead. This option is summarized in table 3.

**Table 3:** Effect of reductions in the number of beds on Key Outcomes

Beds Cut (NumberLeft)	Avg. Percent of Beds Occupied	Percent of Arrivals Who Don't Wait	Avg. Wait Given That the Arrival Must Wait (in days)
0, (96) (no action)	87.5	87.7	1.55

2 (94)	89.3	81.6	1.78

4 (92)	91.3	73.3	2.13

6 (90)	93.3	61.9	2.71

8 (88)	95.4	46.5	3.87

10 (86)	97.7	26.3	7.36

11 (85)	98.8	14.0	14.4

12 or more	100	0	Infinite

Original situation (pre merge)	87.5	66.4	4.11

One can see that two beds can be eliminated without much impact. However, cutting more than ten beds would have large negative effects on the patient's experience with little gain for the facility. Doing so would cause 73.7% of patients to wait, with average wait exceeding seven days.

## Discussion

In the last 100 years, medical care has advanced tremendously, arguably more than in the entire history of humanity prior to that point. Revolutionary treatments have been developed, and people live longer and healthier lives as a result. Improved technology, however, has little power to improve the lives of ordinary citizens unless it is disseminated efficiently and quickly.

Moving from better treatment to better health involves a series of steps; stumbles at any stage can rob patients of the benefits they might gain from better treatments. In some cases, the barrier to better care can be quite simple. For example, it is well established that patients having experienced an acute myocardial infarction should receive an aspirin within 24 hours of hospital admission. More than one in six participants in the Medicare program, however, still do not receive the aspirin [36]. More generally, only half of all patients receive "best practice" treatment for their illness [37]. The failure of providers to follow treatment guidelines is just one example of how knowledge of effective care may not translate into effective care.

Other barriers to care are further removed from the actual experience of care. One such barrier involves the management of resources. Arguably, the technology of treatment has far surpassed the technology of managing the delivery of care. The IOM/NAE report has labeled this disjuncture, the "paradox of American Health Care"[[37], p.11]. This paradox reflects a variety of causes, some beyond the reach of the tools of operations research. One cause is the financing of care fosters a "cottage industry" structure [37]. The resulting fragmentation of care further degrades the quality of care.

As this paper illustrates, one set of tools for improving the delivery of health care can be found in the tools of operations research. We illustrate one of those tools here, queueing models. As is true of the broader set of OR tools, queueing models can accommodate key features of the delivery process of interest, such as the random nature of patient arrivals. Then in turn the model can identify how key outcomes may change in response to policy changes. As we illustrate with an example involving drug treatment, a queueing model can identify the effect of such changes before they are actually implemented in the real world.

One key aspect of the health care system is its complexity, and policy makers want to design and reform the system in a way that fosters goals that can be competing. For example, policy makers have established a regulatory environment that creates barriers between different organizations within the health care system. Efforts to vertically integrate different levels of health care delivery raise antitrust concerns. For example, a hospital may purchase or merge with a provider of home health care, and such integration may improve the coordination between those providers. However, such integration may raise the potential for anticompetitive behavior, such as the steering of patients by the hospital to its provider. Such integration, therefore, may foster the improved management of resources but may have hidden costs.

More generally, these types of complex decisions are the ones for which the benefits of an OR approach are likely the greatest. An OR model can reveal how different choices balance the benefits of vertical integration with the potential market distortion it creates.

## Competing interests

The authors declare that they have no competing interests.

## Authors' contributions

All authors shared in the conceptualization and preparation of the manuscript and the analyses it presents.

## Appendix 1: Equations and the Matlab Code Used to Compute the Performance Measures for the Example Queueing Application Considered in the Paper

Originally, all the calculations were done by using the M-files presented here. During the writing of this paper, the authors came across a piece of software, MCQueue (written by H.C. Tijms and M.C.T. van de Coevering). This piece of software was used to help confirm the results from the M-files contained here. The software can be found at http://staff.feweb.vu.nl/tijms/.

Tijms gives the following (p. 289 eqn 4.5.6)

And

Where:

• *p_j _*is the long run average time spent in state j. (j people in the system)

• λ is the arrival rate of customers

• *D *is the service time for a customer

• *c *is the number of servers

This infinite set of equations is then reduced by the geometric tail approach, P_j _≈ σ * τ^j^, for j ≥ M . (4.5.7) Where *τ *is the unique solution to *e^λD(1-τ)^τ^c ^*= 1. (4.5.8)

We then solve the resultant system using Gaussian elimination.

To achieve this, we use 3 M-files, "pandlcalc," "matmaker," and "taucalc."

Each files purpose:

• "pandlcalc" handles the solving of the systems of equations, and computing the interesting performance characteristics

• "matmaker" creates the matrix which contains the finites system of equations

• "taucalc" calculates tau for use in the other M-files

The inputs are:

• λ, the arrival rate of customers

• *D*, the service time for a customer

• *c*, the number of servers

• tau0, an initial guess for value of tau (it is not important to have a good guess, so long as it is above 1, we use 1.5)

• e, which is an error bounding term used in a few places (1e-6 or 1e-10 is usually good and the execution time is still quick)

• M, is the M for the geometric tail approximation

function output = pandlcalc(lambda, D, c, tau0, e, M)

%this calculates the pvalues and a host of other useful information

% get system of equations and initial tau

mat = matmaker(lambda, D, c, tau0, e, M);

rhs = zeros(M+c+1,1);

rhs(M+c+1, 1) = 1;

tau = taucalc(lambda, D, c, tau0, e);

% calculate initial pvalues

pvals = linsolve(mat, rhs);

%calculate simga, until stable and get resultant pvalues

newsigma = 1;

oldsigma = .1;

while ~(((newsigma/oldsigma) < = (1+e)) && ((oldsigma/newsigma) < = (1+e)))

   oldsigma = newsigma;

   for i = 0:c-1

      newsigma = newsigma + pvals(i+1)*(tau^i-tau^c);

   end

   newsigma = newsigma/(c-lambda*D*tau);

   rhs(M+c+1, 1) = 1-newsigma*(((1/tau)^(M+c))/(1-(1/tau)));

   pvals = linsolve(mat, rhs);

end

%calculate performance measures

numinsys = 0;

numwaiting = 0;

expocc = 0;

proportionnowait = 0;

for i = 0:c-1

   numinsys = numinsys + i*pvals(i+1);

   expocc = expocc + i*pvals(i+1);

   proportionnowait = proportionnowait + pvals(i+1);

end

for i = c:M+c

   numinsys = numinsys + i*pvals(i+1);

   expocc = expocc + c*pvals(i+1);

   numwaiting = numwaiting + (i-c)*pvals(i+1);

end

% calculating waiting time via Little's Law

waittime = numwaiting/lambda;

actualwaits = waittime/(1-proportionnowait);

%{

%alt method

avgtimeinsys = numinsys/lambda

avgwaittimeinsys = avgtimeinsys-D

waittime2 = avgwaittimeinsys/(1-proportionnowait)

%}

%{

%for ploting, if desired

CDFpvals = pvals;

sum = 0;

for i = 1:length(pvals)

   sum = sum+pvals(i);

   CDFpvals(i) = sum;

end

clf;

t = 1:length(pvals);

plot(t, pvals, t, CDFpvals);

figure;

plot(t, pvals);

figure;

plot(t, CDFpvals);

%}

output = [numinsys; expocc; proportionnowait; numwaiting; waittime; actualwaits];

function out = matmaker (lambda, D, c, tau0, e, M)

%this makes the matrix which represents the system of equations for use in

%pandlcalc

%initialize the matrix

mat = zeros(M+c+1, M+c+1);

%fill in the matix

for i = 1:M

   for j = 1:c+i-1

      if j < = c

         mat(i, j) = exp(-lambda*D)*(lambda*D)^(i-1)/factorial(i-1);

      else

         mat(i, j) = exp(-lambda*D)*(lambda*D)^(i-j+c)/factorial(i-j+c);

      end

   end

end

mat = mat-eye(M+c+1, M+c+1);

%continue to fill in for the values which the geometric tail approximation

%will handle

tau = taucalc(lambda, D, c, tau0, e);

for i = M+1:1:M+c

   mat(i, i+1) = tau;

end

for j = 1:M+c+1

   mat(M+c+1, j) = 1;

end

%output

out = mat;

function out = taucalc(lambda, D, c, tau0, e)

%this calculates tau using the recommendation from Tijms p.289

%initialize

taunew = tau0;

tauold = taunew+e+.1;

%iteratively solve

while abs(taunew-tauold) > e

   tauold = taunew;

   taunew = (c*log(tauold))/(lambda*D)+1;

end

%output

out = taunew;

## Pre-publication history

The pre-publication history for this paper can be accessed here:

http://www.biomedcentral.com/1471-2288/10/60/prepub
